# Social inequalities in heat-attributable mortality in the city of Turin, northwest of Italy: a time series analysis from 1982 to 2018

**DOI:** 10.1186/s12940-020-00667-x

**Published:** 2020-11-16

**Authors:** Marta Ellena, Joan Ballester, Paola Mercogliano, Elisa Ferracin, Giuliana Barbato, Giuseppe Costa, Vijendra Ingole

**Affiliations:** 1 Department Environmnetal Sciences, Informatics, and Statistics, Università Ca’Foscari Venezia, 30172 Mestre, Italy; 2 Regional Models and geo-Hydrological Impacts Division, Fondazione Centro Euro-Mediterraneo sui Cambiamenti Climatici (CMCC), Caserta, 81100 Italy; 3 Barcelona Institute for Global Health (ISGlobal), Universitat Pompeu Fabra, CIBER Epidemiología y Salud Pública, 08003 Barcelona, Spain; 4 Regional Epidemiology Unit, ASL TO3 Piedmont Region, 10095 Grugliasco, Italy

**Keywords:** Climate change, Italy, Social inequalities, Summer temperature-attributable mortality, Urban Heat Island

## Abstract

**Background:**

Understanding context specific heat-health risks in urban areas is important, especially given anticipated severe increases in summer temperatures due to climate change effects. We investigate social inequalities in the association between daily temperatures and mortality in summer in the city of Turin for the period 1982–2018 among different social and demographic groups such as sex, age, educational level, marital status and household occupants.

**Methods:**

Mortality data are represented by individual all-cause mortality counts for the summer months between 1982 and 2018. Socioeconomic level and daily mean temperature were assigned to each deceased. A time series Poisson regression with distributed lag non-linear models was fitted to capture the complex nonlinear dependency between daily mortality and temperature in summer. The mortality risk due to heat is represented by the Relative Risk (RR) at the 99th percentile of daily summer temperatures for each population subgroup.

**Results:**

All-cause mortality risk is higher among women (1.88; 95% CI = 1.77, 2.00) and the elderly (2.13; 95% CI = 1.94, 2.33). With regard to education, the highest significant effects for men is observed among higher education levels (1.66; 95% CI = 1.38, 1.99), while risks for women is higher for the lower educational level (1.93; 95% CI = 1.79, 2.08). Results on marital status highlighted a stronger association for widower in men (1.66; 95% CI = 1.38, 2.00) and for separated and divorced in women (2.11; 95% CI = 1.51, 2.94). The risk ratio of household occupants reveals a stronger association for men who lived alone (1.61; 95% CI = 1.39, 1.86), while for women results are almost equivalent between alone and not alone groups.

**Conclusions:**

The associations between heat and mortality is unequal across different aspects of social vulnerability, and, inter alia, factors influencing the population vulnerability to temperatures can be related to demographic, social, and economic aspects. A number of issues are identified and recommendations for the prioritisation of further research are provided. A better knowledge of these effect modifiers is needed to identify the axes of social inequality across the most vulnerable population sub-groups.

**Supplementary information:**

**Supplementary information** accompanies this paper at 10.1186/s12940-020-00667-x.

## Background

### Climate change and heat stress

In a scenario of no or limited adaptation to climate change, extreme temperatures are expected to be one of the main adverse events responsible for additional deaths [1]. Temperature rises already revealed profound heat stress impacts experienced by human populations, with prolonged and more frequent heatwaves and new emerging threats to public health [2, 3]. In 2018, over 220 million persons over the age of 65 were additionally exposed to heatwaves compared to a climatological baseline, and of those the majority lived in urban areas [4]. In fact, cities experience twice as many heat days than surrounding areas and by the end of the twenty-first century this number is projected to increase 10-fold [5]. The effects around the globe are not evenly distributed [6] and the impacts can differ by city and between suburban areas since the conditions within cities are not equal in all their parts [1, 7, 8]. Overall, severe increases in temperature are projected in Europe, with the highest levels of warming expected in Mediterranean regions during summer seasons [3, 9–11]. Of those, Italy is the country with the highest heat-related effects on daily mortality considering summer temperatures [12]. In particular, studies over urban areas located in the northern regions of Italy highlighted how these specific areas reached the greatest excess in mortality due to heat in the past [13–15] and they are characterised by a strong positive association between the number of daily emergency visits [16] and daily mean air temperatures.

### Urban population susceptibility

Associations between heat and mortality are generally unequal across different aspects of society, and several studies have documented the importance of the context specific risks, which can vary by spatial, climatological and population characteristics [17–19]. Inter alia, factors influencing the population vulnerability to temperature are related to demographic, social, and economic aspects [20]. According to the World Health Organization (WHO), a better knowledge of these effect modifiers is needed to identify the axes of inequality across the most vulnerable population groups [2, 21]. Nowadays, in regard to gender inequality, some studies have identified higher mortality rates in women compared to men [4, 22–24] across different ages [25, 26], while some others observed men to be more at risk under heat stress conditions [27, 28]. Evidence about the relevance of age to the increase of heat related mortality have been observed in different cities [4, 21, 29]. Qualitative [30] as well as quantitative studies [31–34] on heat stress agree that the temperature-related mortality risk increases in the elderly. Moreover, according to an investigation by Leone et al. [29] that considered different age-group thresholds in different Mediterranean cities, none of the studies provide evidence of direct health effects among young persons [29].

### The importance of socio-economic inequalities

Regarding the socio-economic factors, statistical associations have been found between mortality and socio-economic drivers in the case of vulnerability to heat, such as education [34–38], marital status [39, 40], employment status [41–43], as well as household structure factors [30, 44]. An adequate health adaptation response requires an assessment of the vulnerability of populations as a baseline analysis, but most of the environmental epidemiological studies rely exclusively on the available statistical data and do not have access to more precise information [4]. To the extent of our knowledge, while some studies have identified the effect modification of education in the heat-mortality relationship in a European urban context [21], just a few focussed on marital status and on the relationship between the household structure characteristics and the mortality risk, with no insight into how these socio-economic factors influence mortality under heat stress conditions. Therefore, in this study, the first objective was to develop a distributed lag non-linear model (DLNM) to estimate the non-linear and delayed effects of summer temperatures on mortality in the city of Turin for the period 1982–2018, looking at social inequality factors such as education, marital status and number of household occupants as well as to linkages between variables. Second, the research defines the attributable risk due to heat for the entire 37-year observational study, through the calculation of attributable fractions and attributable numbers differentiated by summer months, sex and socio-economic groups.

## Methods and materials

### Study area

Turin (45°6′ 58″ N and 7°44′ 33″ E) is located in the north-west part of Italy and it is the fourth largest Italian urban area with a population of 875698 inhabitants [45]. The city is located 800 ft above sea level and despite the climate predominantly being characterised by dry summers and mild wet winters (Mediterranean), the presence of the Alpine mountain range and the Superga hills favours a limited circulation of the foehn winds, conferring to Turin a complex mosaic of microclimates.

### Data sources

With the aim of defining the relationship between summer temperatures and mortality, individual mortality records and individual-specific socioeconomic status for the period of 1982–2018 were collected. Following the Guidelines on Warning-System Development [2], a long time frame have been selected in order to track the changes of heat occurrences into the historical context over the study area. Only summer deaths (15th of May until the 15th of September) were considered in this analysis (according to the local heat wave bulletin). Data on individual all-cause mortality and socio-economic status were obtained from a dynamic population-based database, called Turin Longitudinal Study (TLS) [45]. As a measure of individual socio-economic position, the educational level, the marital status and the number of household occupants of the deceased were used [46]. Following the original structure of the dataset [45], the educational level was categorized into three groups (“no more than primary education”, “secondary education”, “high school or more”) and the marital status were categorised into four groups (“un-married”, “married”, “widower”, “separated and divorced”). The household occupant’s variable into two main groups (“alone” and “not alone”). For practical reasons, the “separated and divorced” category of the marital status variable were merged into one singular group, and the age variable was divided into four groups (“0–64”, “65–74”, “75–84” and “85+”). Therefore, the social inequalities were analysed diversifying the analysis by sex and by sub-groups (see Table 1), looking at cross-linkages between variables.

**Table 1:** Number of deaths, MMT (CI 95%) and 99th RR (CI 95%) per socio-economic variables

Categories	Sub-categories	N° of deaths	MMT (°C)	95% CI	RR at P99	95% CI
**Mortality by**	**Men**	53909	16.2	(12.1, 18.0)	1.56	(1.45, 1.67)
**Age-group**	0–64 years old	12151	14.7	(9.0, 20.4)	1.32	(1.13, 1.55)
	65–74 years old	13006	14.5	(9.0, 19.2)	1.44	(1.23, 1.69)
	75–84 years old	17677	18.0	(11.2, 20.0)	1.53	(1.37, 1.71)
	85+ years old	11075	15.5	(15.0, 18.2)	2.04	(1.76, 2.38)
**Education**	No more than primary school	28417	15.8	(9.0, 18.2)	1.64	(1.49, 1.80)
	Secondary school	14564	18.8	(9.0, 21.1)	1.36	(1.20, 1.54)
	High school or more	10624	13.9	(9.0, 18.4)	1.66	(1.38, 1.99)
**Marital status**	Married	38704	16.3	(11.1, 18.3)	1.54	(1.41, 1.67)
	Separated and divorced	2479	20.6	(9.0, 26.2)	1.39	(1.07, 1.81)
	Unmarried	6062	11.7	(9.0, 19.7)	1.63	(1.20, 2.23)
	Widower	6630	16.0	(9.0, 19.8)	1.66	(1.38, 2.00)
**Household occupants**	Alone	9489	17.9	(9.0, 21.4)	1.61	(1.39, 1.86)
	Not alone	44187	15.7	(9.8, 17.9)	1.53	(1.42, 1.66)
**Mortality by**	**Women**	56046	17.2	(15.6, 18.3)	1.88	(1.77, 2.00)
**Age-group**	0–64 years old	6799	14.4	(9.0, 32.1)	1.26	(1.00, 1.58)
	65–74 years old	8422	16.7	(9.0, 19.1)	1.69	(1.43, 1.99)
	75–84 years old	18277	17.1	(11.4, 19.0)	1.90	(1.71, 2.11)
	85+ years old	22548	17.9	(16.3, 19.0)	2.13	(1.94, 2.33)
**Education**	No more than primary school	36827	17.2	(15.4, 18.3)	1.93	(1.79, 2.08)
	Secondary school	12698	18.3	(13.8, 20.1)	1.83	(1.62, 2.07)
	High school or more	6252	16.1	(9.0, 19.7)	1.69	(1.39, 2.05)
**Marital status**	Married	17892	16.4	(9.0, 18.9)	1.71	(1.52, 1.92)
	Separated and divorced	2069	16.9	(9.0, 32.1)	2.11	(1.51, 2.94)
	Unmarried	7777	17.4	(9.0, 19.8)	1.87	(1.60, 2.20)
	Widower	28267	17.5	(15.7, 18.7)	1.97	(1.81, 2.14)
**Household occupants**	Alone	24055	17.9	(15.9, 19.1)	1.88	(1.72, 2.05)
	Not alone	31451	16.3	(12.6, 18.0)	1.89	(1.74, 2.06)

Exposures to daily mean temperature in summer were assigned to each deceased case based on a temperature time series built through an arithmetic mean of mean temperature values related to each of the nine grid cells within the boundaries of the city. Climate data were obtained from the MESCAN-SURFEX system (5.5 km resolution), which consists of a surface re-analysis dataset using an optimal interpolation algorithm for 2 m ambient temperature, available for each day at 00 (H), 06, 12 and 18 UTC [47].

### Statistical analysis

The statistical analysis was performed in 2 stages. In the first stage, a generalised linear model with standard quasi-Poisson regression was used to estimate the association between heat and mortality, reported as Relative Risks (RR). In the second stage, a DLNM model was applied to examine the complex non-linear and delayed dependencies between daily summer temperature and mortality values. The analyses were stratified by sex, age-group, education, marital-status and household occupants of the deceased.

Firstly, the short-term association of daily summer temperatures and all-cause mortality was investigated. A time series Poisson regression were fitted for modelling seasonality and long-time trends through a standard quasi-Poisson model to account for the over-dispersion of daily death records. With reference to prior research, seasonal trends were controlled by using a natural cubic B-spline of the day-of-the-season with 2 degree of freedom per year [23]. The latter parameter was permitted to vary from 1 year to another through the inclusion of an interaction between the applied natural cubic spline and the year. A natural cubic B-spline of time with 1 degree of freedom per decade was included to control for the long-term pattern [48]. Finally, the day-of-the-week factor was inserted in the time series quasi-Poisson regression as an indicator variable.

The key feature of the second-stage analysis is the investigation of the non-linear and delayed effect which specifies the temporal dependency between summer temperature and mortality on the scale of lag, here defined as exposure-lag-response association. To capture this complex non-linear dependency, a DLNM was included through the definition of a cross-basis, obtained by the combination of two functions describing respectively the exposure-response and the lag-response association [49–51]. The exposure–response curve was modelled consisting of a quadratic B-spline with 1 internal knot placed at the 90th percentile of the temperature distribution. For the lag-response curve, a natural cubic spline with 2 equally spaced knots on the log scale was applied. The effect of daily summer temperatures on mortality up to 7 days of lag to capture the overall temperature effect and adjusting for any potential harvesting [52] was assessed. The model choices were based on the quasi-Akaike Information Criterion (qAIC) and on modelling choices from previous works [23, 50, 53, 54]. The final algebraic representation of the model is:

1 $$ Log\left({\mu}_t\right)=\alpha + cb+{dow}_t+ ns\left({dos}_t\; df=2\right): factor\left({year}_t\right)+ ns\left({date}_t, df=1\; per\;{decade}_t\right) $$

where *μ*_t_ is the expected number of deaths at the day of observation *t*, *cb* is the cross-basis matrix produced by DLNM, *dow* is the categorical variable for the day of the week and *ns* specifies the natural cubic B-spline for day-of-the-season and for the year/summer respectively. At this stage of the analysis, the temperature at which risk of overall mortality is at minimum was identified, here called minimum mortality temperature (MMT). This value was calculated with its confidence intervals (95% CI) through the use of a parametric bootstrap method proposed by Tobías et al. [55]. MMT values for all mortality counts and by socio-economic categories were calculated to capture the social inequality differences in the temperature-mortality associations. The mortality risk due to heat was represented by the RR at the 99th percentile of daily summer temperatures. Then, the attributable fraction (AF) and the attributable number (AN) of deaths by summer months, considering the “total heat” (for all days exceeding the MMT), the “moderate heat” (between the MMT and the 97th percentile of daily summer temperatures) and the “extreme heat” period (exceeding the 97th percentile) were calculated. These estimates provided the relative excess measure and the absolute excess measure due to the exposure to different levels of summer temperatures [56]. For the attributable values, we only showed results for the June, July and August (JJA) months.

All statistical analyses were performed with *R software (version 3.6.0)* through the use of the *dlnm* package, developed by Gasparrini [51].

## Results

**Fig. 1:**
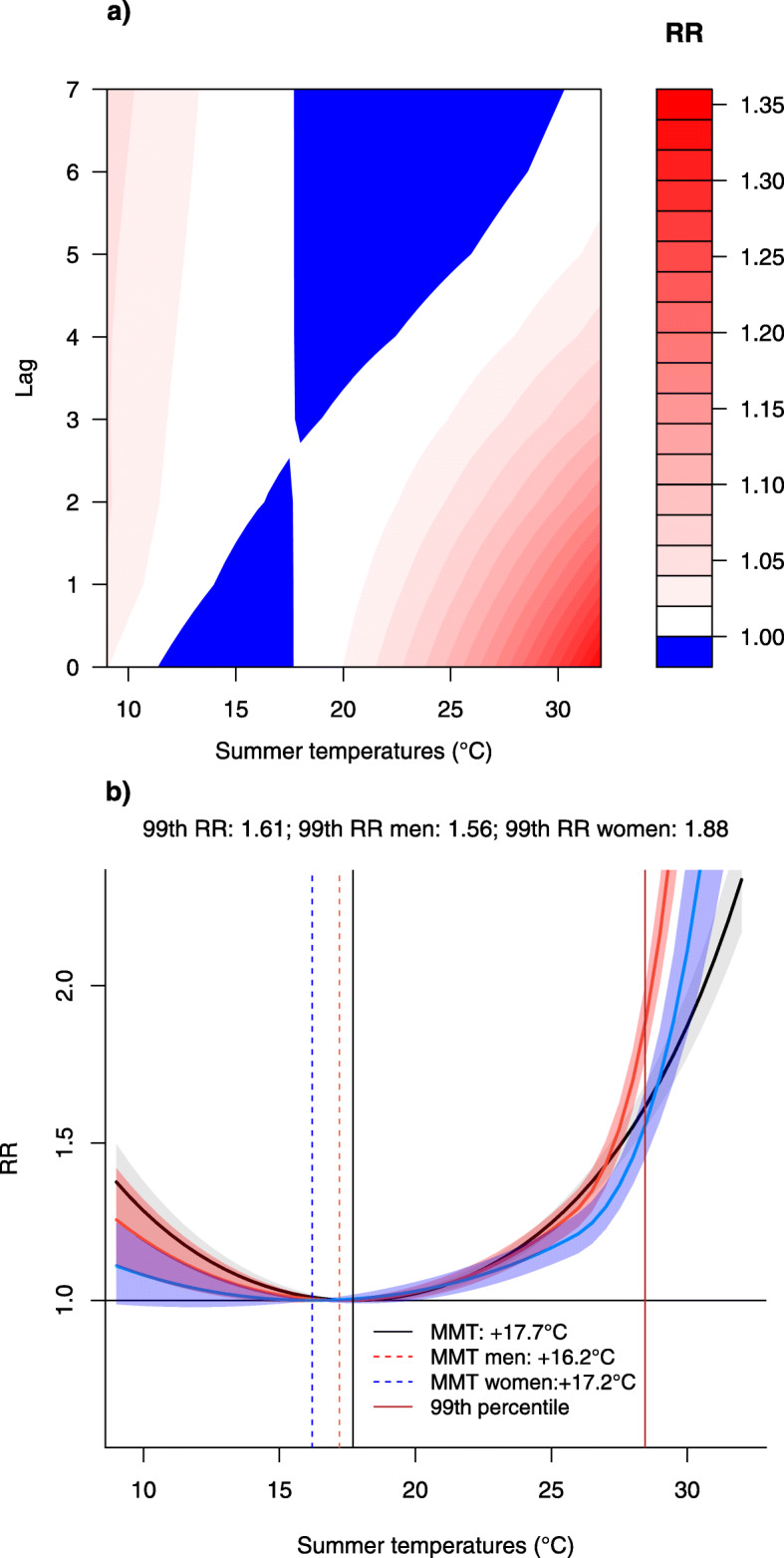
The exposure-response relationship between summer temperatures and all-cause mortality. **a** Contour plot with reference at the 99th percentile Relative Risk (RR). **b** Overall cumulative temperature-mortality association between summer temperatures and mortality in the city of Turin by sex (RRs in solid lines and 95% CI in shaded colours)

In the 37-year observational study, the mean daily temperature range was between 10° and 32 °C, with the 50th, 95th and the 99th percentile respectively equal to 22°, 26.9° and 28.5 °C. The daily average mortality count was 24, with a minimum number of deaths per day equal to 8 and a maximum number of deaths per day equal to 83. Figure 1 depicts the effect of daily summer temperatures on all-cause mortality by sex. From the contour plot (Fig. 1(a)) it emerges that the risks decrease with increasing lead times, with higher RR under hot temperature conditions. On the other hand, the cumulative exposure-response association differentiated by sex (Fig. 1(b)) shows how the overall association between daily mean temperatures and mortality during summer months follow a U-shaped curve in both sexes, with representative MMT and RR values.

The dataset contained 109955 deaths, of which 53909 men (49%) and 56046 women (51%). In Table 1 a summary of the characteristics of the study population was presented. As mentioned in the previous section, a representative MMT and a corresponding 99th RR for each sub-group differentiated by sex was detected. 95% confidence intervals (CI) are also provided*.* The temperature-mortality relationship through the visualization of the overall cumulative plots, which highlights a non-linear “U shaped association for all the available socio-economic categories, was summarised (see A1.1-A1.4 Figs in Additional file 1 for overall cumulative plots by sex and socioeconomic sub-group).

### Age

Mortality records were divided into four categories based on the age of the deceased person at the time of death: “0–64”, “65–74”, “75–84”, and “85+” years old. In the youngest groups (“0–64” and “65–74” years), the number of women was slightly lower (15221) than the number of men (25157), while at the oldest groups (“75–84” and “85+”) the number was higher in women (40825) than in men (28752). In fact, women live longer and die older than men [57, 58], therefore gender and age are here interlinked. The RR at the 99th percentile grows with age in both sexes, with a RR in the age group “85+” equal to 2.04 (95% CI: 1.76–2.38) in men and 2.13 (95% CI:1.94–2.33) in women. In all the sub-groups the RRs were positive and significant, with higher RRs in women compared to men.

### Education

When considering education, we classified the groups into “no more than primary school”, “secondary school” and “high school or more”. The “no more than primary school” was the sub-group with the highest number of observations (28417 for men, 36827 for women), while “high school or more” was the most restricted one (10624 for men, 6252 for women). Results in Table 1 display a significant effect of summer temperatures in all the sub-groups, with a significantly higher effect for women in respect to men. The most significant effects of heat for men was found in the “high school or more” and in the “no more than primary school” sub-groups, with a RR equal to 1.66 (95% CI: 1.38, 1.99) and to 1.64 (95% CI: 1.49, 1.80) respectively. On the other hand, in women a higher effect was found in the “no more than primary school”, with a RR equal to 1.93 (95% CI: 1.79–2.08). Furthermore, while for women the most significant effect reflects the lowest level of education and decreases with increasing education, in men it became less straightforward. Therefore, cross-linkages between education, gender and age seem to play an important role in the latter sub-category.

### Marital status

The marital status categorisation was divided into the following sub-groups: “un-married”, “married”, “separated and divorced” and “widower”. The most substantial sub-category of observations for men corresponded to “married” (38704), while for women it was to “widower” (28267). In addition, the sex gap was also visible under this socio-economic driver, which implies an interconnection with the above variable and the age category. The RRs show a higher effect on women than men. In men, it was found that the most significant effects are in the “widower” and in the “unmarried” sub-groups, with a RR equal to 1.66 (95% CI: 1.38, 2.00) and to 1.63 (95% CI: 1.20, 2.23) respectively. On the other hand, in women a higher effect it was found in the “separated and divorced” sub-group as well as in the “widower” sub-groups, which corresponded to a RR of 2.11 (95% CI: 1.51, 2.94) and to 1.97 (95% CI: 1.81, 2.14). In general, all the RR estimates were significant.

### Household occupants

Under this categorisation, all results were diversified by sex and significant. In particular, the most significant effect for men was the one that refers to people who lived “alone”, that corresponded to a RR equal to 1.61 (95% CI: 1.39, 1.86). On the contrary, in women, the RRs between the two sub-categories were almost the same, with the most significant effect in the “not alone” group, which is equal to 1.89 (95% CI: 1.74, 2.06). Moreover, people living “not alone” were the most prevalent under this category, with 44187 records in men and 31451 records in women.

Results of AFs and ANs were analysed by sex, looking at the most significant estimates for “moderate heat”, “extreme heat” and “total heat” conditions. Comparing the hottest 3 months of the time series (June, July and August), the highest AFs and ANs were obtained for “total heat” temperatures in July (AN tables provided in A2.1 Table, Additional file 2). Table 2 shows how AF estimates were higher for women compared to men. In fact, the total AF for men was 13% (95% CI: 10, 17) with AN equal to 1836 (95% CI: 1365, 2349), while the total AF for women was 17% (95% CI: 15–20) with AN equal to 2544 (95% CI: 2113, 2946). The highest AFs and ANs were obtained among the “85+” age-groups, with AF equal to 23%(95% CI: 15, 31) in men and to 21% (95% CI: 16, 24) in women. When considering education, the highest AFs were related to the “high school or more” sub-groups for men (18% (95% CI: 7, 30)), while for women was related to the “no more than primary school” (18% (95% CI: 15, 22)), in accordance with the RRs. On the contrary, when considering ANs, it was found that the highest values was in the category “no more than primary school”, which corresponded to 1070 (95% CI: 681, 1429) in men and to 1821 (95% CI: 1478, 2157) in women.

**Table 2:** Mortality attributable fraction due to moderate, extreme and all temperatures by sex, with 95% confidence intervals (CI)

Attributable fraction of deaths (AF) with CI 95%		JUNE			JULY			AUGUST		
		Moderate	Extreme	Total heat	Moderate	Extreme	Total heat	Moderate	Extreme	Total heat
**Mortality by:**	**Men**	7.0% (4.0, 9.0)	1.0% (0.0, 1.0)	8.0% (4.0, 10.0)	11.0% (8.0, 15.0)	2.0% (2.0, 2.0)	13.0% (10.0, 17.0)	9.0% (6.0, 12.0)	3.0% (2.0, 3.0)	12.0% (9.0, 15.0)
**Age-groups**	0–64 y	5.0% (−4.0, 13.0)	0% (0, 0)	5.0% (−4.0, 13.0)	9.0% (−3.0, 17.0)	1.0% (0, 2.0)	10.0% (−3.0, 19.0)	8.0% (− 1.0, 17.0)	1.0% (0, 2.0)	9.0% (−1.0, 19.0)
	65–74 y	7.0% (−2.0, 15.0)	0% (0, 1.0)	7.0% (−2.0, 16.0)	11.0% (− 2.0, 19.0)	2.0% (1.0, 2.0)	13.0% (−1.0, 21.0)	9.0% (−1.0, 17.0)	2.0% (1.0, 3.0)	11.0% (0, 20.0)
	75–84 y	5.0% (2.0, 8.0)	1.0% (1.0, 1.0)	6.0% (3.0, 9.0)	9.0% (4.0, 13.0)	2.0% (2.0, 2.0)	11.0% (6.0, 15.0)	7.0% (2.0, 11.0)	3.0% (2.0, 3.0)	10% (4.0, 14.0)
	85+ y	13.0% (6.0, 19.0)	1.0% (1.0, 1.0)	14.0% (7.0, 20.0)	20.0% (12.0, 27.0)	3.0% (3.0, 4.0)	23.0% (15.0, 31.0)	17.0% (9.0, 24.0)	5.0% (4.0, 5.0)	22.0% (13.0, 29.0)
**Education**	No more than primary school	8.0% (4.0, 11.0)	1.0% (0, 1.0)	9.0% (4.0, 12.0)	12.0% (7.0, 17.0)	2.0% (2.0, 3.0)	14.0% (9.0, 20.0)	10.0% (6.0, 15.0)	3.0% (2.0, 3.0)	13.0% (8.0, 18.0)
	Secondary school	4.0% (1.0, 6.0)	0% (0, 1.0)	4.0% (1.0, 7.0)	6.0% (1.0, 11.0)	1.0% (1.0, 2.0)	7.0% (2.0, 13.0)	4.0% (0, 8.0)	2.0% (1.0, 3.0)	6.0% (1.0, 11.0)
	High school or more	11.0% (−1.0, 21.0)	1.0% (1.0, 1.0)	12.0% (0, 22.0)	16.0% (5.0, 27.0)	2.0% (2.0, 3.0)	18.0% (7.0, 30.0)	14.0% (3.0, 24.0)	3.0% (2.0, 4.0)	17.0% (5.0, 28.0)
**Marital status**	Married	7.0% (5.0, 10.0)	0% (0, 0)	7.0% (5.0, 10.0)	13.0% (9.0, 16.0)	2.0% (1.0, 2.0)	15.0% (10.0, 18.0)	11.0% (7.0, 14.0)	2.0% (2.0, 2.0)	13.0% (9.0, 16.0)
	Separated and divorced	4.0% (0, 8.0)	1.0% (0, 1.0)	5.0% (0, 9.0)	7.0% (−1.0, 14.0)	1.0% (−1.0, 2.0)	8.0% (−2.0, 16.0)	5.0% (−3.0, 12.0)	1.0% (−3.0, 3.0)	6.0% (−6.0, 15.0)
	Unmarried	8.0% (1.0, 14.0)	0% (0, 1.0)	8.0% (1.0, 15.0)	13.0% (4.0, 21.0)	2.0% (1.0, 3.0)	15.0% (5.0, 24.0)	11.0% (2.0, 20.0)	2.0% (1.0, 3.0)	13.0% (3.0, 23.0)
	Widower	8.0% (4.0, 12.0)	1.0% (1.0, 1.0)	9.0% (5.0, 13.0)	14.0% (6.0, 20.0)	3.0% (2.0, 3.0)	17.0% (8.0, 23.0)	11.0% (5.0, 17.0)	3.0% (2.0, 4.0)	14.0% (7.0, 21.0)
**Household occupants**	Alone	4.0% (1.0, 8.0)	1.0% (0, 1.0)	5.0% (1.0, 9.0)	7.0% (1.0, 13.0)	2.0% (2.0, 3.0)	9.0% (3.0, 16.0)	6.0% (−1.0, 11.0)	3.0% (2.0, 4.0)	9.0% (1.0, 15.0)
	Not alone	8.0% (5.0, 11.0)	1.0% (0, 1.0)	9.0% (5.0, 12.0)	12.0% (8.0, 16.0)	2.0% (2.0, 2.0)	14.0% (10.0, 18.0)	10.0% (6.0, 14.0)	2.0% (2.0, 3.0)	12.0% (8.0, 17.0)
**Mortality by:**	**Women**	8.0% (7.0, 10.0)	1.0% (1.0, 1.0)	9.0% (8.0, 11.0)	14.0% (12.0, 17.0)	3.0% (3.0, 3.0)	17.0% (15.0, 20.0)	12.0% (9.0, 14.0)	4.0% (4.0, 5.0)	16.0% (13.0, 19.0)
**Age-groups**	0–64 y	8.0% (−4.0, 18.0)	0% (0, 0)	8.0% (−4.0, 18.0)	12.0% (−2.0, 23.0)	1.0% (0, 2.0)	13.0% (−2.0, 25.0)	10.0% (−4.0, 22.0)	1.0% (−1.0, 2.0)	11.0% (−5.0, 24.0)
	65–74 y	10.0% (4.0, 15.0)	1.0% (0, 1.0)	11.0% (4.0, 16.0)	15.0% (8.0, 22.0)	3.0% (2.0, 3.0)	18.0% (10.0, 25.0)	13.0% (6.0, 20.0)	3.0% (2.0, 4.0)	16.0% (8.0, 24.0)
	75–84 y	7.0% (4.0, 10.0)	1.0% (1.0, 1.0)	8.0% (5.0, 11.0)	12.0% (8.0, 17.0)	3.0% (3.0, 4.0)	15.0% (11.0, 20.0)	10.0% (5.0, 14.0)	4.0% (4.0, 5.0)	14.0% (9.0, 18.0)
	85+ y	10.0% (7.0, 12.0)	1.0% (1.0, 1.0)	11.0% (8.0, 13.0)	17.0% (13.0, 20.0)	4.0% (3.0, 4.0)	21.0% (16.0, 24.0)	13.0% (10.0, 17.0)	6.0% (5.0, 6.0)	19.0% (15.0, 23.0)
**Education**	No more than primary school	9.0% (7.0, 11.0)	1.0% (1.0, 1.0)	10.0% (8.0, 12.0)	15.0% (12.0, 18.0)	3.0% (3.0, 4.0)	18.0% (15.0, 22.0)	13.0% (10.0, 16.0)	4.0% (4.0, 4.0)	17.0% (14.0, 20.0)
	Secondary school	6.0% (3.0, 9.0)	1.0% (1.0, 1.0)	7.0% (4.0, 10.0)	10.0% (6.0, 15.0)	3.0% (2.0, 3.0)	13.0% (8.0, 18.0)	8.0% (3.0, 13.0)	5.0% (4.0, 5.0)	13.0% (7.0, 18.0)
	High school or more	9.0% (1.0, 17.0)	1.0% (0.0, 1.0)	10.0% (1.0, 18.0)	14.0% (3.0, 23.0)	2.0% (2.0, 3.0)	16.0% (5.0, 26.0)	11.0% (0, 20.0)	4.0% (3.0, 5.0)	15.0% (5.0, 25.0)
**Marital status**	Married	8.0% (6.0, 11.0)	0% (0, 1.0)	9.0% (6.0, 12.0)	14.0% (10.0, 18.0)	2.0% (2.0, 2.0)	16.0% (12.0, 20.0)	12.0% (8.0, 15.0)	3.0% (2.0, 3.0)	15.0% (10.0, 18.0)
	Separated and divorced	16.0% (3.0, 27.0)	1.0% (0, 1.0)	17.0% (3.0, 28.0)	25.0% (9.0, 38.0)	3.0% (2.0, 4.0)	28.0% (11.0, 42.0)	21.0% (6.0, 35.0)	5.0% (3.0, 7.0)	26.0% (9.0, 42.0)
	Unmarried	9.0% (5.0, 12.0)	1.0% (1.0, 1.0)	10.0% (6.0, 13.0)	16.0% (11.0, 22.0)	3.0% (2.0, 4.0)	19.0% (13.0, 26.0)	14.0% (9.0, 18.0)	4.0% (3.0, 4.0)	18.0% (12.0, 22.0)
	Widower	10.0% (8.0, 12.0)	1.0% (1.0, 1.0)	11.0% (9.0, 13.0)	18.0% (15.0, 21.0)	3.0% (3.0, 4.0)	21.0% (18.0, 25.0)	15.0% (12.0, 17.0)	4.0% (4.0, 4.0)	19.0% (16.0, 21.0)
**Household occupants**	Alone	8.0% (6.0, 10.0)	1.0% (1.0, 1.0)	9.0% (7.0, 11.0)	14.0% (11.0, 17.0)	3.0% (3.0, 3.0)	17.0% (14.0, 20.0)	11.0% (8.0, 14.0)	4.0% (4.0, 5.0)	15.0% (12.0, 19.0)
	Not alone	9.0% (6.0, 12.0)	1.0% (1.0, 1.0)	10.0% (7.0, 13.0)	15.0% (11.0, 19.0)	3.0% (3.0, 4.0)	18.0% (14.0, 22.0)	13.0% (8.0, 16.0)	4.0% (4.0, 4.0)	17.0% (12.0, 20.0)

In accordance with the 99th RR estimates, the highest AFs for the marital status category were the “widower” group (AF: 17% (95% CI: 8, 23)) for men, while for women it was the “divorced and separated” group (28% (11, 42)). In regard to AN, it was found to be the “widower” group that had the highest significant value (AN: 1581 (95% CI: 1332, 1801)), which is the largest class within this category for women. When dealing with the household occupants’ category, the highest statistically significant AFs obtained was obtained for the “not alone” groups in both sexes, which corresponded to 14% (95% CI: 10, 18) in men and to 18% (95% CI: 14, 22) in women. Finally, the statistically significant highest ANs for the prominent sub-groups (“not alone”) ((men: 1611 (95% CI: 1151, 2067), (women: 1492 (95% CI: 1119, 1831)).

Multiple sensitivity analyses were performed, changing key modelling decisions in order to check the consistency of the results and to investigate whether day-to-day changes in the number of deaths are explained by changes in summer ambient temperatures. Starting with the full-year analysis, focusing on the summer period only, since our objectives were on summer heat. For the standard quasi-Poisson model analyses, first the type of function of time to capture the long-time trend and seasonality of the mortality data was analysed, based on Bhaskaran [48]. Having defined the spline function to capture seasonal patterns in a way that is allowed to vary from 1 year to the next, the number of knots was modified in order to identify the best spline to use. Later, in the specification of the cross-basis functions to define the DLNM, a consistent amount of analyses was applied to understand (i) the exposure response function to use (ns or bs), (ii) the percentile of temperature to focus on and the related number of knots to use, and, finally, (iii) the lag number to use in order to take into account the short-term effect of heat as well as the harvesting effect. To better focus the final choice, the qAIC from different models was compared (see A3.1-A3.3 Figs in Additional file 3).

Consequently, it is believed that the parameters used in the final model of this study can adequately capture the main effect of summer temperatures on mortality.

## Discussion

Results of our study contribute to the overall cumulative associations of summer temperatures and mortality comprehension for the city of Turin over the 37-year observational study, reporting results stratified by demographic and socio-economic drivers. In particular, to the best of knowledge of those involved in this study, this is the first investigation to assess comprehensively social inequalities in relation to the heat-health nexus, looking at sex, age, educational level, marital status and household occupants at the same time. This allow to provide a more specific overview on how different drivers can affect heat stress in a South-European urban context. The achieved results strongly support the hypothesis that the different sub-categories that refer to each social variable can negatively or positively affect the risk of heat mortality, contributing significantly to the variation of the mortality fraction attributable to heat. In general, results suggested that the effect of heat on mortality largely varied by each analysed category with higher RRs for women compared to men for each sub-category of interest. Moreover, cross-linkages between demographic and socio-economic drivers were also visible. According to the state-of-the-art literature, the mortality risk grows with age in both sexes, and the study found a statistically significant association for all the ages. With regards to education, significant effects of heat in all the groups was found and, contrary to prior expectations, while for women risk values were higher for lower educational level and decreased as education increased, in men the stronger effects corresponded to those with higher “formal” education as well as to those with lower educational levels. Mortality risk ratios by marital status were higher for those who lived alone (e.g. unmarried, separated and divorced and widower) than for married people, in both sexes. Results on household occupants consistently indicate a strong association among men who lived alone, while for women results were equivalent for the two analysed groups.

### Sex

All-causes mortality was assessed by sex, and it was found that women were systematically more at risk than men under heat stress conditions. These results are in line with many studies, which identify higher mortality rates for women compared to men [4, 22, 57, 58], specifically in the European context [21, 23, 25, 26]. This discrepancy may arise from differences in response to thermal stress due to physiological characteristics in body temperature regulation [59, 60] as well as pre-existing socio-demographic characteristics in the inhabited society [11, 26], such as the lower social condition that characterises elderly women, which often live alone due to longer life expectancy compared to men.

### Age

Age is one of the main personal factors that determines heat vulnerability [22], and is the reason for having investigated this factor by sub-groups and sex. In all the age-groups a positive significant association between summer temperatures and mortality was found. The RRs at the 99th percentile increased with age and RRs were consistently higher in women than men. In fact, women live longer and die older, while men die younger [58, 61]. Therefore, the relationship between the age and the sex variable here is consistent. These results extended previous hypothesis based on the evidence that heat stress increases the susceptibility of the elderly to hot temperatures with advancing age [27, 30, 41, 58]. Intrinsic changes in the thermoregulatory system, the presence of pre-existing diseases, the use of certain medication together with the social conditions that characterise older people (e.g. single occupancy) makes this category more susceptible to heat events than other sub-groups [2].

### Education

With regard to education, a significant effect of heat on mortality in all the educational groups was observed, with higher RRs for women compared to men. In men the highest significant effects were seen in the higher level of education (“high school or more”) as well as in the lower once (“no more than primary school”). In contrast, risk values for women were higher for lower educational level and decreased as education increased. In this context, several studies underline how an individual’s educational background may influence the health outcomes [21, 62, 63]. Very often, it is assumed that the higher the educational level, the more appropriate individual adaptation measures are applied during periods of heat stress [35, 61]. These hypotheses are in line with the results achieved for women, but it is not the case for men. Some evidence from Turin suggested that the achieved results in this study are a combination of two phenomena. The first one is related to the mortality conditions observable among sexes in the City of Turin. Costa et al. [45, 57] highlighted how men characterized by low socio-economic conditions corresponded to high rates of premature deaths. In fact, men die younger, particularly if less educated [64–66]. This imply that individuals with lower levels of education are associated to a higher risk of mortality in younger ages, reason why the “no more than primary school” corresponded to a high RR. On the other side, following the same reasoning, deaths attributable among older men can be associated to higher levels of education, reason why the “high school or more” sub-group present a high RR for men. The second phenomenon is the value attributed to education in different social careers of men and women over the last 40 years. The northern cities of Italy have had many experiences of migration, especially from the regions of the south, that not only affects the life of migrants, but more generally changes the life horizons of all Turin citizens [67, 68]. Therefore, to understand the social and employment structure of Turin, it is important to take into account the phenomena of social stratification created by the different waves of migration. Men from the south are often associated with very low education, condition that make them suitable for a premature mortality. In addition, the remaining male population from the south with higher educational qualifications were discriminated from the native population of Turin, implying fewer career opportunities [45]. To the best of knowledge of those involved in this study, the novel results presented here partly contrast those of previous works; instead suggesting that for the population of Turin the association of mortality risk with heat is stronger in higher levels of education (“high school or more”) as well as in lower level of education (“no more than primary school”) for men, while for women stronger association have been found in lower levels of education (“no more than primary school”).

### Marital status

Results of RR by marital status highlighted a significant effect in all groups. Overall, RR were higher for the “unmarried”, “separated and divorced” and “widowed”, than for those “married” in both sexes with higher RRs for women than men. Over the years, several studies analysed the statistical association between marital status and mortality [64, 65, 69–71], however, very few studies focused on the relationship between marital status and mortality under heat stress events [39, 66, 72], due to limited data availability. Of those studies, the marital status is often used as a proxy for the family structure [39, 73] or as a proxy for social isolation [40, 74]. The most recent epidemiological and demographic research shows a beneficial effect of marriage on the health of the spouses [75–78]. In fact, in accordance with a study over French related to the 2003 heat wave [66], results generally suggested higher impacts for unmarried, widowed, separated and divorced people rather than those who were married. Marriage offers a ‘protective effect’ for health; it encourages healthier lifestyle [69], it discourages risk taking behaviour, it increases more favourable societal attitudes [79] and it may increase material well-being [64]. Therefore, ‘protective effect’ of the family on health manifests itself through lower risk of death, lower likelihood of experiencing phases of depression or anxiety, lower health problems, benefits that are often more pronounced for men. From the analyses, AF values for men were higher among the “widowed” group. The consequences of the stress of losing a spouse through divorce or widowhood can have long-term consequences for both men and women’s mental and physical health [45]. This effect was evident among women, who were found to have a higher AF values in the “widowed” as well as in the “separated and divorced” category. Previous studies suggested how the death of the spouse for widowers [80, 81], or the dissolution of the marriage for the separated and divorced [19, 66, 82, 83], can be a dramatic and stressful event, with considerable health consequences [45]. In fact, benefits of married life also seem to accumulate during the marriage, with a long-term beneficial effect that could come from the mutual support. Moreover, in contrast with the literature state-of-the-art which recognises that “single” men are more at risk than women [65, 69], the study’s findings show higher mortality risks for women. This result could be attributable to the fact that women live longer than men in our case study, and therefore the probability of being alone in advanced age for women is higher [57]. This is a novel in the literature, since it is the first time that these results have been found in studies related to mortality and temperature association during summer periods.

### Number of household occupants

With regard to the number of household occupants, in men the results indicate a stronger evidence in individuals who lived alone at the time of death (in accordance with the conclusions found for the previous category). On the other hand, in women the RR differences between the two sub-categories were minimal. Among socio-economic drivers relevant for increased heat vulnerability, the “number of household occupants” (also called household structure in the literature) is relevant to take into account the degree at which an individual is integrated into networks and social relationships [17, 80, 84]. In fact, as Seebaß [30] highlighted, “the more social interactions a person has, the lower their perception of heat stress is”. As the analysis by Aubrecht and Özceylan [35] shows, living alone can be a significant indicator that possibly resulting in fewer contacts with family and friends, increasing vulnerability and eventually mortality under heat stress condition [35]. In contrast, some other studies found a not significant correlation of living alone with increasing mortality [37, 44], highlighting how the type of social contacts a person has as well as the frequency of interacting with these contacts are more important in subjective heat stress [30]. Costa et al. [45] pointed out how generally differences in social inequalities decreases with age. Therefore, considering that women live longer than men, it can be assumed that the discrepancies between living “alone” and “not alone” decrease with age in women, aspect that emerged from the analyses.

Hence, the two categories mentioned above (marital status and household occupants) are logically interlinked [85]. In fact, some case studies used the marital status as a proxy for the family structure [39, 40] while some others used the household structure variable as a proxy for social isolation [31, 36, 39, 44]. In this research, both were used to see the differences of RRs by sub-categories and sex. In men the RR was higher for single-person households, in accordance with the marital status results, while for women RRs between “alone” and “not alone” were almost equivalent, demonstrating a minimum deviation from the previous analysed variables. Therefore, the fact that being socially integrated reduces mortality risk due to heat can be partially confirmed in this study.

The key strength of this study is the extension of results seen in previous investigations on temperature and mortality association (37 years observational study), by looking at age-groups, education, marital status and household occupants, stratified by sex, which were previous knowledge gaps in the literature. It was possible to run these analyses thanks to the availability of a continuous record linkage between the mortality register and the municipal census. Another strength of the study is that in order to have mean temperature values for the whole study period, an evaluation of the uncertainty comparing the mean temperature time series of the used dataset with other two climate datasets was provided [84]. This comparison highlighted that the selected dataset reproduces well the climatology obtained from the local meteorological station for the city of Turin. These processes allowed for the largest daily time series - of 37 years in the European urban context - to be developed. The main limitations of the study are related to two different aspects. First, information on specific causes of deaths were not available for the whole study period, reason why the present research did consider all-causes mortality. However, datasets on all-causes mortality are often employed in the heat-health nexus research due to both the lack of available data and to the absence of a uniform definition for heat-related death [31, 54, 85, 86]. Second, the range of possibilities and studies with ozone (O_3_) or particular matter (PM_2.5–10_) has not been fully sampled or explored, due to the lack of data for the reference period. In addition, the spatial distributions of vulnerabilities were not taken into account, which could have helped the understanding as to how sensitive populations are distributed within the sub-urban area under study. This issue will be further assessed in future research, through the adoption of a time-stratified case-crossover design followed by the application of geostatistical models using geo-coded mortality and environmental data. This will allow policymakers to better understand the district- and neighbourhood-level vulnerability within the urban environment. Finally, it was decided to not include the possibility of potential temporal changes in effect estimates due to adaptation of the population (or action taken). This aspect is crucial to understand more precisely if there are any dynamic impacts (such as adaptation) in the data over the entire time-frame and if there is a relative contribution of interannual temperature anomalies and year-to-year climate variability to the evolution of each attributable fractions related to every socio-economic driver. In fact, the significance of each considered variable in relation to the health of the population could have changed over the time. Therefore, in order to be able to locate the precise steps of the changes of predictive capacity for each analysed variable and to analyse the relative risk variations of cause-specific mortality across the whole range of summer temperatures, a dedicated scientific study with the use of time-varying DLNMs is under development. This evidence can give greater detail and aid in the understanding of how population risks change over time, and how external policies influence these adaptation processes.

## Conclusions

This study proposes a distributed lag non-linear model for characterising the non-linear and delayed effects of summer temperatures on mortality in the city of Turin for the period 1982–2018. The study shows that demographic, social and economic drivers such as sex, age, education, marital status and household occupants play an important role in determining the most vulnerable groups within a population, also looking at cross-linkages between variables. This is important since having better knowledge of these effect modifiers is necessary to identify the axes of inequalities across the most vulnerable population sub-groups and to therefore contribute towards relevant policy risk mitigation suggestions.

## Supplementary information

**Additional file 1.** Overall cumulative plots by sex and socioeconomic sub-group.

**Additional file 2.** Attributable number of deaths by demographic and socio-economic drivers.

**Additional file 3.** Sensitivity analyses for modelling choices.

## Data Availability

The climate dataset analysed during the current study is available in the Copernicus data store repository [https://cds.climate.copernicus.eu/cdsapp#!/dataset/reanalysis-uerra-europe-single- levels?tab = form]. The mortality and socio-economic datasets that support the findings of this study are available from the Regional Epidemiology Unit of ASL TO3, but restrictions apply to the availability of these data, which were used under license for the current study and so are not publicly available. Data are however available from the authors upon reasonable request and with permission from the Regional Epidemiology Unit of ASL TO3.

## Abbreviations

CI: confidence intervals
DLNM: distributed lag nonlinear model
MMT: minimum mortality temperatures
RR: relative risk
