# Supplementary material for: Social inequalities in heat-attributable mortality in the city of Turin, northwest of Italy: a time series analysis from 1982 to 2018
Source: Environ Health. 2020 Nov 16;19:116. doi: 10.1186/s12940-020-00667-x (PMC7667731; doi:10.1186/s12940-020-00667-x)
Supplement: Supplementary file 1 — Additional file 1. Overall cumulative plots by sex and socioeconomic sub-group. [file 12940_2020_667_MOESM1_ESM.docx]

**Additional file 1**

**Overall cumulative plots by sex and socioeconomic sub-group**

- *
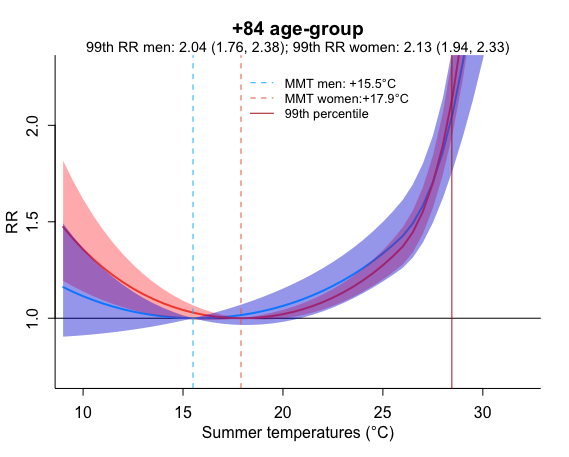

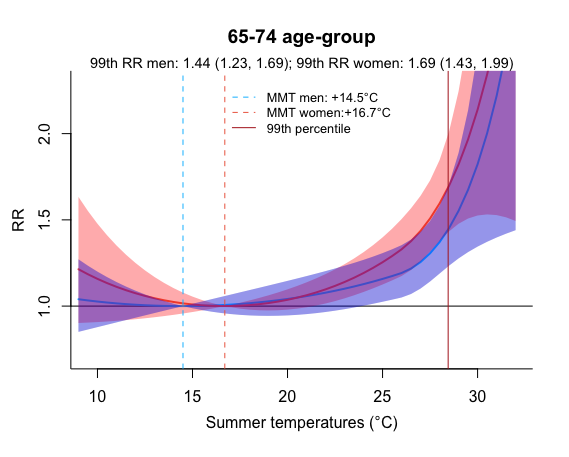

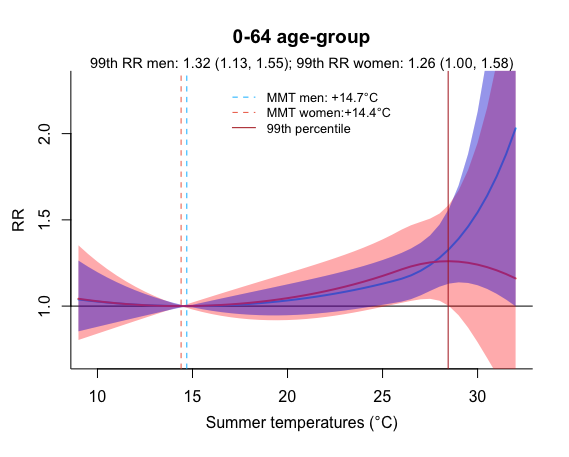
***Overall cumulative plots by sex and age-groups**

*
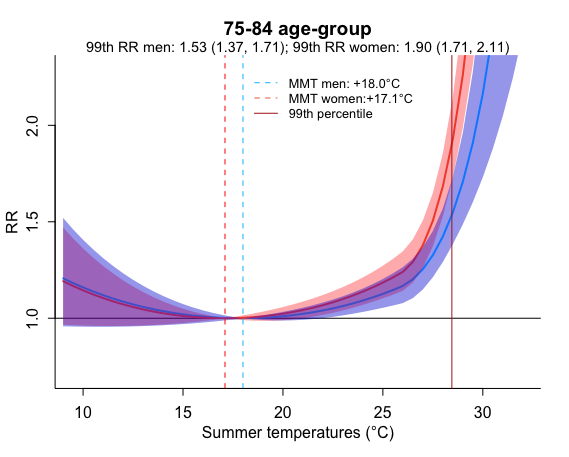
*

**Fig. A1.1** **Age-group by sex**

Overall cumulative plots showing the RR curves for age-groups by sex: men (blue) and women (red)(RRs in solid lines, MMT in dotted lines and 95% CI in shaded colours).

- **Overall cumulative plots by sex and education**

**
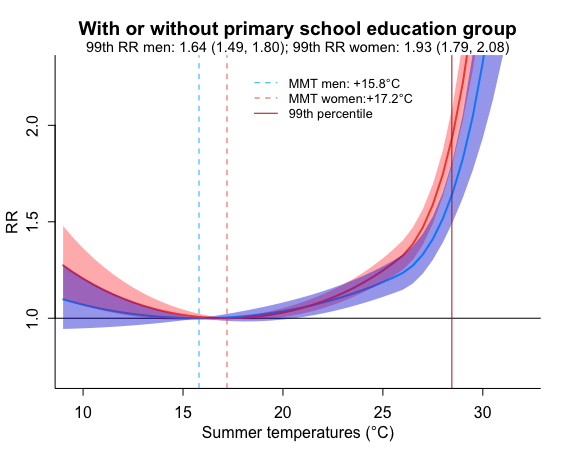

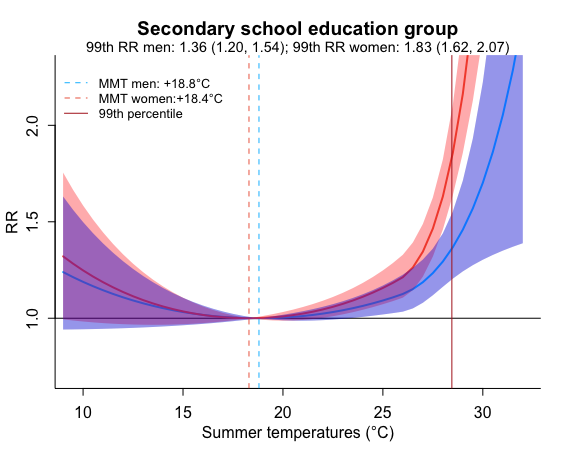
**

**
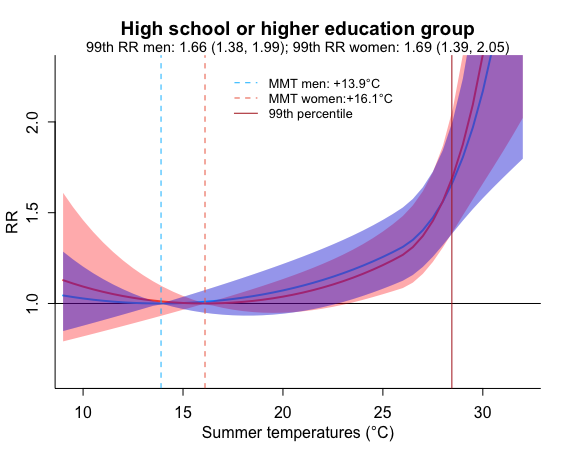
**

**Fig. A1.2**  **Education by sex**

Overall cumulative plots showing the RR curves for educational level by sex: men (blue) and women (red) (RRs in solid lines, MMT in dotted lines and 95% CI in shaded colours).

- **Overall cumulative plots by sex and marital status**

*
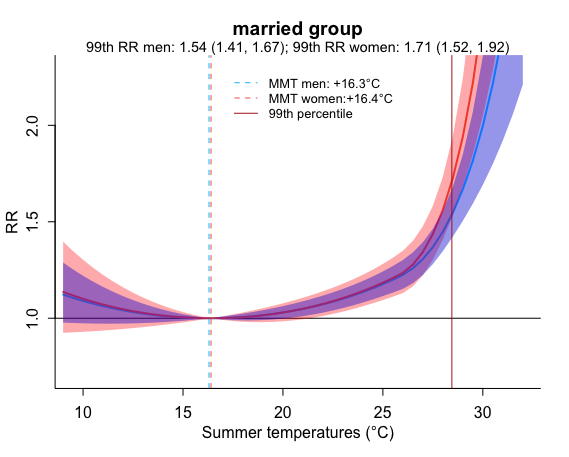

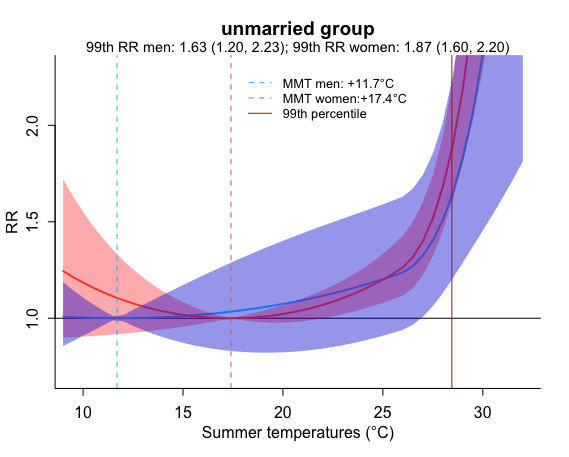

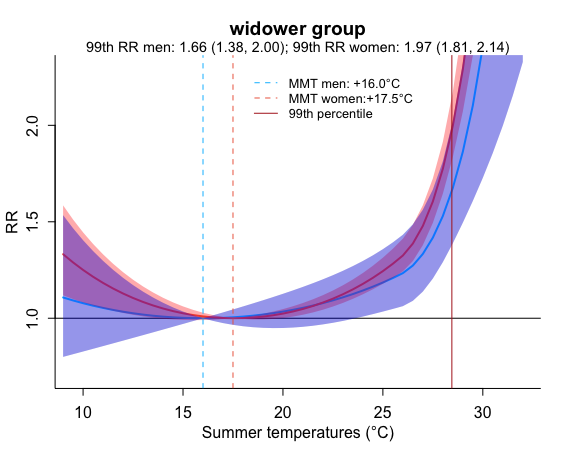

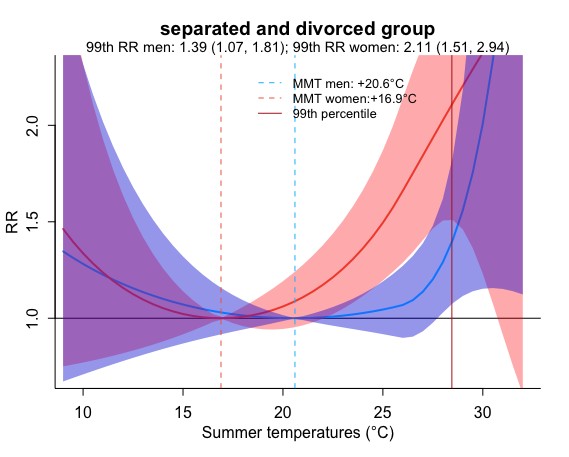
*

**Fig. A1.3** **Marital status by sex**

Overall cumulative plots showing the RR curves for marital status by sex: men (blue) and women (red) (RRs in solid lines, MMT in dotted lines and 95% CI in shaded colours).

- **Overall Cumulative Plots by sex and household occupants**

**
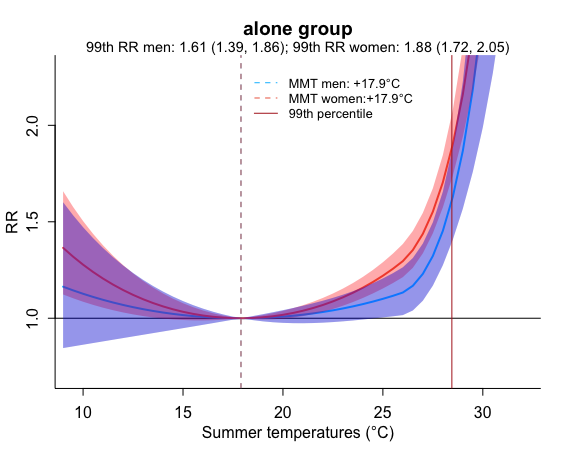
**

**
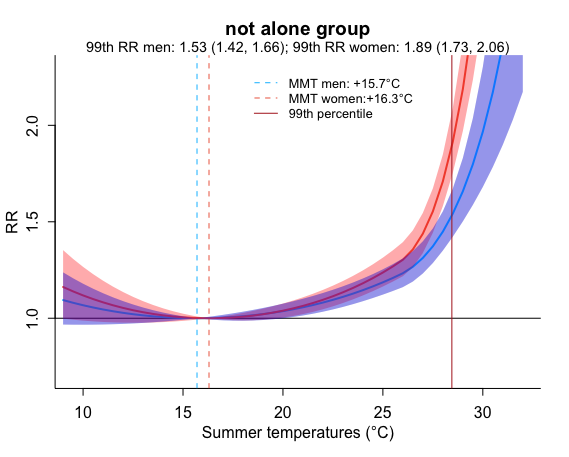
**

**Fig. A1.4 Household occupants by sex**

Overall cumulative plots showing the RR curves for household occupants by sex: men (blue) and women (red) (RRs in solid lines, MMT in dotted lines and 95% CI in shaded colours).
