# Supplementary material for: Social inequalities in heat-attributable mortality in the city of Turin, northwest of Italy: a time series analysis from 1982 to 2018
Source: Environ Health. 2020 Nov 16;19:116. doi: 10.1186/s12940-020-00667-x (PMC7667731; doi:10.1186/s12940-020-00667-x)
Supplement: Supplementary file 2 — Additional file 2. Attributable number of deaths by demographic and socio-economic drivers. [file 12940_2020_667_MOESM2_ESM.docx]

**Additional file 2**

**Attributable number of deaths by demographic and socio-economic drivers**

**Table A2.1** Death counts attributable to moderate, extreme and all temperatures by sex, with 95% confidence intervals (CI)

| **Attributable number of deaths (AN)**  **with CI 95%** | | **JUNE** | | | **JULY** | | | **AUGUST** | | |
| --- | --- | --- | --- | --- | --- | --- | --- | --- | --- | --- |
|  |  | **Moderate** | **Extreme** | **Total heat** | **Moderate** | **Extreme** | **Total heat** | **Moderate** | **Extreme** | **Total heat** |
| **Mortality by:** | **Men** | 886  (527, 1214) | 74  (65, 83) | 960  (592, 1297) | 1547  (1114, 2021) | 289  (251, 328) | 1836  (1365, 2349) | 1245  (823, 1714) | 348  (296, 397) | 1593  (1119, 1711) |
| **Age-groups** | 0 – 64 y | 142  (-78, 355) | 6  (2, 10) | 148  (-76, 365) | 278  (-10, 530) | 42  (16, 61) | 320  (-6, 591) | 252  (-48, 532) | 39  (12, 60) | 290  (-36, 592) |
|  | 65 – 74 y | 224  (-41, 457) | 15  (9, 20) | 238  (-32, 477) | 369  (57, 673) | 54  (30, 74) | 421  (87, 747) | 290  (-37, 577) | 66  (36, 89) | 353  (-1, 666) |
|  | 75 – 84 y | 219  (98, 329) | 28  (22, 33) | 247  (120, 362) | 392  (177, 585) | 96  (75, 115) | 489  (252, 700) | 291  (94, 479) | 118  (84, 148) | 409  (178, 627) |
|  | 85+ y | 361  (170, 526) | 26  (21, 31) | 393  (191, 560) | 556  (348, 758) | 93  (75, 111) | 649  (423, 869) | 479  (271, 677) | 136  (107, 160) | 604  (378, 837) |
| **Education** | No more than primary school | 531  (274, 789) | 41  (34, 46) | 572  (308, 835) | 896  (536, 1229) | 174  (145, 200) | 1070  (681, 1429) | 734  (360, 1054) | 191  (151, 227) | 925  (511, 1281) |
|  | Secondary school | 129  (35, 207) | 14  (10, 18) | 143  (45, 225) | 247  (70, 412) | 53  (34, 71) | 300  (104, 483) | 163  (10, 298) | 72  (40, 100) | 235  (50, 398) |
|  | High school or more | 273  (-18, 528) | 20  (13, 26) | 293  (-5, 554) | 441  (118, 703) | 64  (41, 83) | 505  (159-786) | 395  (68, 689) | 85  (56, 111) | 480  (124, 800) |
| **Marital status** | Married | 683  (455, 888) | 46  (39, 53) | 729  (494, 941) | 1252  (911, 1559) | 173  (142, 199) | 1425  (1053, 1758) | 1046  (735, 1336) | 186  (149, 221) | 1232  (884, 1557) |
|  | Separated and divorced | 23  (-3, 45) | 2  (-0, 4) | 25  (-3, 49) | 43  (-12, 88) | 9  (0, 16) | 52  (-12, 104) | 30  (-17, 68) | 9  (-5, 19) | 39  (-22, 87) |
|  | Unmarried | 115  (12, 206) | 7  (3, 10) | 122  (15, 216) | 208  (62, 333) | 28  (14, 40) | 236  (76, 373) | 170  (33, 283) | 33  (18, 46) | 203  (51, 329) |
|  | Widower | 128  (57, 190) | 14  (10, 17) | 142  (67, 207) | 250  (135, 355) | 50  (34, 62) | 300  (169, 417) | 187  (71, 290) | 47  (29, 62) | 234  (100, 353) |
| **Household occupants** | Alone | 93  (-3, 174) | 16  (11, 20) | 109  (8, 194) | 177  (2, 319) | 62  (44, 77) | 239  (46, 396) | 135  (-10, 270) | 83  (59, 104) | 218  (49, 374) |
|  | Not alone | 815  (468, 1163) | 58  (50, 66) | 873  (518, 1229) | 1386  (962, 1809) | 225  (189, 258) | 1611  (1151, 2067) | 1134  (717, 1527) | 259  (208, 298) | 1393  (925, 1825) |
| **Mortality by:** | **Women** | 1120  (863, 1390) | 111  (102, 119) | 1331  (965, 1509) | 2079  (1687, 2442) | 465  (426, 504) | 2544  (2113, 2946) | 1621  (1254, 1965) | 591  (541, 639) | 2212  (1795, 2604) |
| **Age-groups** | 0 – 64 y | 127  (-50, 287) | 4  (0, 7) | 131  (-50, 294) | 208  (-34, 419) | 21  (3, 35) | 239  (-31, 454) | 164  (-60, 368) | 15  (-10, 34) | 179  (-70, 402) |
|  | 65 – 74 y | 194  (80, 289) | 12  (9, 15) | 206  (89, 304) | 340  (178, 499) | 59  (41, 73) | 399  (219, 572) | 274  (123, 413) | 58  (37, 74) | 332  (160, 487) |
|  | 75 – 84 y | 305  (158, 435) | 32  (28, 37) | 337  (186, 472) | 598  (372, 818) | 160  (137, 180) | 758  (509, 998) | 455  (263, 655) | 199  (174, 221) | 645  (437, 876) |
|  | 85+ y | 528  (387, 660) | 64  (58, 70) | 592  (438, 730) | 969  (769, 1175) | 224  (200, 247) | 1,193  (914, 1422) | 765  (576, 950) | 324  (290, 355) | 1089  (847, 1305) |
| **Education** | No more than primary school | 784  (576, 969) | 74  (68, 80) | 848  (644, 1049) | 1488  (1175, 1792) | 333  (303, 365) | 1821  (1478, 2157) | 1175  (894, 1464) | 382  (339, 416) | 1557  (1233, 1880) |
|  | Secondary school | 189  (99, 283) | 24  (20, 28) | 213  (119, 311) | 341  (180, 493) | 91  (74, 106) | 432  (254, 599) | 259  (110, 393) | 148  (121, 168) | 407  (231, 561) |
|  | High school or more | 132  (4, 241) | 11  (7, 14) | 143  (11, 255) | 230  (52, 39) | 42  (27, 54) | 272  (79, 450) | 170  (17, 301) | 61  (39, 79) | 231  (56, 380) |
| **Marital status** | Married | 353  (230, 469) | 23  (18, 27) | 376  (248, 496) | 667  (461, 861) | 99  (78, 116) | 766  (539, 977) | 522  (344, 684) | 114  (85, 138) | 636  (419, 822) |
|  | Separated and divorced | 75  (18, 124) | 3  (2, 4) | 78  (20, 128) | 137  (60, 199) | 17  (10, 23) | 154  (70, 222) | 117  (32, 176) | 28  (15, 38) | 145  (47, 214) |
|  | Unmarried | 162  (98, 224) | 17  (13, 20) | 179  (101, 244) | 337  (223, 445) | 65  (50, 77) | 402  (273, 522) | 273  (170, 374) | 72  (54, 86) | 345  (224, 460) |
|  | Widower | 685  (551, 808) | 64  (58, 70) | 749  (609, 878) | 1332  (1109, 1526) | 249  (223, 275) | 1581  (1332, 1801) | 1036  (864, 1224) | 283  (251, 312) | 1319  (1115, 1536) |
| **Household occupants** | Alone | 457  (322, 581) | 50  (44, 55) | 507  (366, 636) | 891  (649, 1229) | 199  (175, 223) | 1090  (824, 1352) | 670  (475, 876) | 270  (237, 297) | 940  (712, 1173) |
|  | Not alone | 700  (466, 937) | 61  (54, 67) | 761  (520, 1004) | 1227  (884, 1537) | 265  (235, 294) | 1492  (1119, 1831) | 992  (665, 1288) | 316  (276, 351) | 1308  (941, 1639) |
