# Supplementary material for: Social inequalities in heat-attributable mortality in the city of Turin, northwest of Italy: a time series analysis from 1982 to 2018
Source: Environ Health. 2020 Nov 16;19:116. doi: 10.1186/s12940-020-00667-x (PMC7667731; doi:10.1186/s12940-020-00667-x)
Supplement: Supplementary file 3 — Additional file 3. Sensitivity analyses for modelling choices. [file 12940_2020_667_MOESM3_ESM.docx]

**Additional file 3**

**Sensitivity analyses for modelling choices**


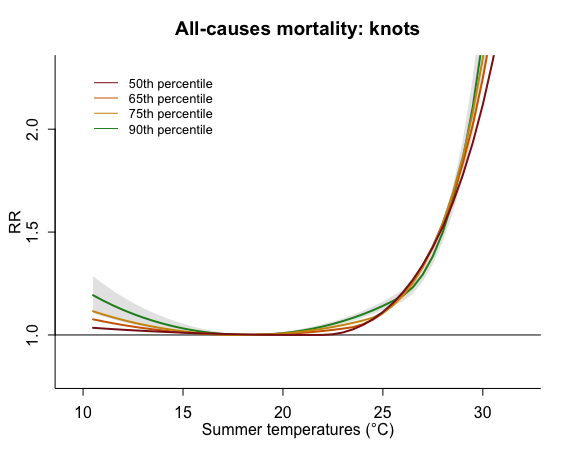


**Fig A3.1.** Knots for exposure-response


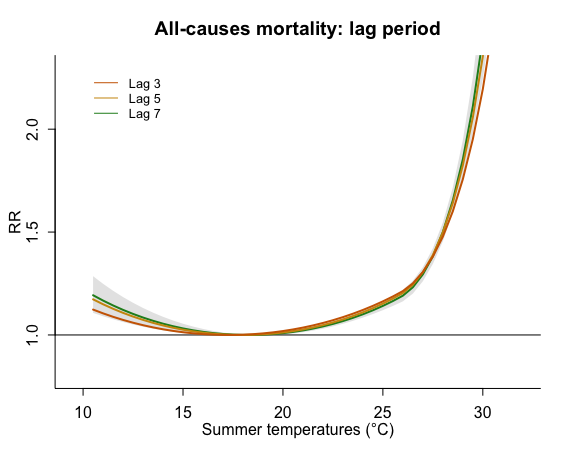


**Fig A3.2.** Lag period

**
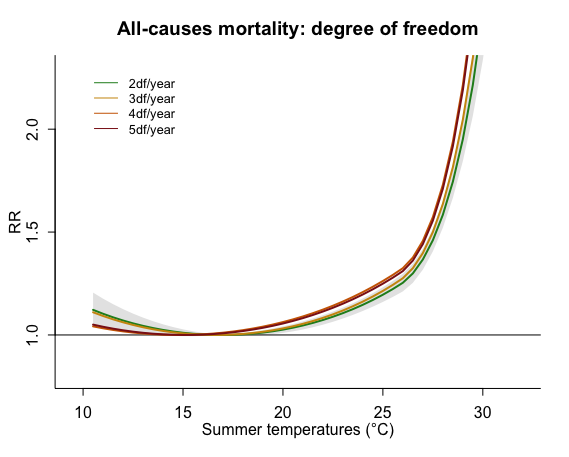
**

**Fig A3.3.** Degrees of freedom for seasonal trend
